# From Injury to Independence: Investigating the Impact of Hand Burn Severity on Functional Outcomes in Children and Adolescents Followed for 24 Months After Injury—A Prospective Cohort Study

**DOI:** 10.3390/ebj7010017

**Published:** 2026-03-18

**Authors:** Ingrid Parry, Cameron Ward, Jeffrey Fine, David G. Greenhalgh, Michelle A. James, Katharine M. Hinchcliff

**Affiliations:** 1 Shriners Children’s Northern California, Sacramento, CA 95817, USA; dggreenhalgh@health.ucdavis.edu (D.G.G.); mjames@shrinenet.org (M.A.J.); 2 California Northstate University College of Medicine, Elk Grove, CA 95757, USA; cameron.ward9580@cnsu.edu; 3 Department of Public Health Sciences, School of Medicine, University of California, Sacramento, CA 95817, USA; jrfine@health.ucdavis.edu; 4 Department of Surgery and Orthopaedic Surgery, University of California San Diego, San Diego, CA 92093, USA; khinchcliff@health.ucsd.edu

**Keywords:** burns, scars, pediatric, hands, hand burn severity score, HABS, burn outcome questionnaire, BOQ, PROMIS, upper extremity function

## Abstract

**Highlights:**

**What are the main findings?**

- Higher Hand Burn Severity Scores (HABS) at the time of injury were significantly associated with worse upper extremity functional outcome in children, particularly in the early post-burn period (2–6 months).
- Higher HABS was independently associated with poorer patient-reported function on PROMIS-UE8 across the full 24-month follow-up period.

**What are the implications of the main findings?**

- HABS may serve as a useful early risk stratification tool to identify pediatric patients at higher risk for functional impairment, enabling targeted monitoring and intervention.
- Early rehabilitation planning and resource allocation may be particularly important for children with higher HABS scores to optimize long-tern upper extremity outcomes.

**Abstract:**

Background: Hand burns are common in children and can result in long-term functional impairment. The Hand Burn Severity (HABS) score is an anatomy-specific measure of hand burn severity, but its relationship to functional outcomes in pediatric patients has not been well defined. The purpose of this study was to determine whether HABS, measured at the time of injury, is associated with longitudinal upper extremity functional outcomes in children. Methods: We conducted a 24-month prospective longitudinal study of children aged 2–18 years with hand burns. Burn severity was determined using HABS at enrollment, and outcomes were measured using the Burn Outcomes Questionnaire (BOQ) and the eight-item Patient-Reported Outcomes Measurement Information System-Upper Extremity (PROMIS-UE8). Repeated-measures linear regression models evaluated associations between HABS and outcomes over time, adjusting for age, dominant- and bilateral-hand involvement, and surgery. Results: A total of 119 children with 165 hand burns were enrolled. Higher HABS scores, indicating more severe injury, were significantly associated with lower BOQ upper extremity function domain scores, indicating poorer health, at 2- and 6-months post burn, but not at later time points. Higher HABS scores were independently associated with lower patient-reported PROMIS-UE8 scores, indicating poorer health, across the 2-year follow-up period. Conclusions: These findings indicate that higher HABS scores at the time of injury are associated with poorer upper extremity outcomes, particularly in the early post-burn period, suggesting that HABS may help identify children who could benefit from closer monitoring or early rehabilitation planning.

## 1. Introduction

As part of normal development, children use their hands to explore. This increases their risk of hand injuries, including burns to the hands [1,2,3]. Hands are involved in about half of all burn injuries in children, and they are one of the most frequent areas to develop deformity and contracture after injury [4,5,6]. Although many pediatric hand burns heal uneventfully, scar contractures resulting from 2nd- and 3rd-degree burns impact growth, development, and function, which can result in substantial long-term deformity and disability [7,8,9].

Identifying children at high risk of poor functional outcomes early in treatment can guide appropriate interventions, inform resource utilization, and prevent functional problems from developing [10]. Such early risk identification could inform the timing and type of surgical and nonsurgical interventions needed. The extent and depth of the burn often determine the severity of the injury and the necessary surgical and nonsurgical treatment. Typically, burn severity is determined by the total body surface area (TBSA) burned and measured over the entire body. However, the hands make up only 5% of the TBSA, and burns to the hands often result in diminished function out of proportion to the magnitude of the TBSA. The complex anatomy and critical functional role of the hands indicate that TBSA is not the optimal metric to predict outcomes after hand burns. Burn depth and the location of the injury within the hand are likely more closely related to the injury’s functional impact [11,12].

### 1.1. Hand Burn Severity (HABS) Score

The HABS score was developed to quantify the impact of burn extent, depth, and location on the hands [13]. HABS divides each hand into three anatomical zones: distal, middle and proximal. A score of 0 to 3 is assigned to each zone on the dorsal and volar areas, yielding a possible score of 18 for each hand (9 dorsally, 9 volarly). HABS scores have been shown to be associated with the need for surgery after a hand burn [13]. Bache et al. reported that a HABS score of 6 was associated with a 50% chance of undergoing surgical excision, and a score of 11 or more was associated with a 100% likelihood. The HABS score has demonstrated more sensitivity and specificity than burn depth alone when determining the need for surgery [13]. HABS has been investigated in relation to longer-term outcomes in adult burn populations using the Burnt Hand Outcome Tool (BHOT), which measures hand function and disability in adult burn survivors [14]. Adult burn survivors with hand burns who scored six or higher on HABS reported significantly poorer outcomes up to one year after injury [14]. Although the items on the BHOT are not suitable for children, there is a need to determine if, in pediatric burn survivors, the HABS is meaningfully related to functional outcomes. Establishing this relationship in children and adolescents is crucial to improving prognostic accuracy and enabling tailored interventions that can ultimately improve outcomes and reduce long-term disability for pediatric burn survivors.

### 1.2. Patient-Reported Outcomes Measurement Information System—Upper Extremity, 8-Item (PROMIS-UE8)

Patient-Reported Outcomes Measurement Information System (PROMIS) questionnaires include patient- and proxy-reported functional outcomes for children and adolescents. These standardized measures, developed by the National Institutes of Health, aim to compare self-reported function scores for adults and children living with chronic conditions with those of the general population [15]. Evidence from PROMIS profiles in burn populations supports the overall integrity of using PROMIS instruments with this population [16,17]. Pediatric and Proxy PROMIS tools have been shown to be reliable and valid for use with pediatric burn survivors and their caregivers as well [16,18]. An abstract of a study including 163 pediatric burn survivors demonstrated that individual burn characteristics significantly impacted self-reported PROMIS upper extremity function (PROMIS-UE8) [19]. However, this study did not specifically evaluate hand burns.

### 1.3. Burn Outcomes Questionnaires (BOQs)

BOQ tools are burn-specific measures recommended for assessing health-related quality of life outcomes in children and adolescents [20]. They are self-reported outcome tools that assess dimensions of physical and psychosocial functioning in children and adolescents aged 18 or younger with burn injury [21,22]. The BOQs take into account normal expected growth in physical and mental development using age-specific reference groups [23]. Psychometric properties of the BOQs have been established based on the experience of over 1200 burn survivors and their families, and the tool has been shown to be reliable and valid when assessing burn outcomes as reported by children, adolescents, and parents of children with burns [24,25,26]. Although studies using the BOQs have shown that the presence of a hand burn increases the risk of poorer outcomes for children (refs. [4,27]), no studies have examined whether the severity of hand burns, as characterized by anatomy-specific metrics, relates to BOQ-measured functional recovery. This gap in the literature limits our ability to identify which pediatric patients with hand burns, despite total TBSA burned, are most vulnerable to long-term impairment.

Our study directly addressed this gap by investigating how hand burn severity impacts the functional recovery trajectory of children and adolescents who sustain burn injuries involving their hands. Specifically, we examined associations between HABS at the time of injury and outcome scores for BOQ domains related to upper extremity function (BOQ-UE) and PROMIS-UE function at 2, 6, 12, and 24 months after burn injury. Our hypothesis was that hand burn severity, as measured by HABS, is associated with self-reported and proxy-reported pediatric outcomes after hand burns. We hope that understanding how burn severity indices that account for the specific anatomy and function of the hands related to the recovery trajectory of children after hand burns could enable earlier, more personalized decision-making regarding appropriate plans of care.

## 2. Materials and Methods

### 2.1. Study Design and Population

This study was a single-site, prospective, longitudinal cohort observational study of pediatric patients with hand burns of varying severity. Patients between the ages of 2 and 18 years with a diagnosis of a 2nd- or 3rd-degree burn to at least one hand were enrolled and followed for 2 years post injury. Patients were excluded if they had isolated fingertip burns, cognitive impairment, pre-injury abnormal hand deformity or functional limitation, true electrical burn, or compartment syndrome or nerve injury affecting the burned hand. This study is part of a larger study measuring performance-based outcomes so younger children were not enrolled. The first subject was enrolled in April 2018. Due to enrollment delays during the COVID-19 pandemic, the last subject received final follow-up testing in April 2023.

### 2.2. Outcome Measures and Timeline

HABS was completed by the same primary burn surgeon for all subjects upon study enrollment using direct observation of the injury and photographs or burn diagrams taken at the time of injury, which were standardized for the study. The patient completed the age appropriate BOQ (primary outcome) and PROMIS-UE outcome questionnaires if they were 8 years or older, or a proxy caregiver completed them if the patient was younger than 8 years. The questionnaires were completed in person or via telephone at approximately 2, 6, 12, and 24 months after burn injury.

The BOQ instrument generates domain-specific scores that quantify functional, physical, and psychosocial outcomes after burn injury. For this analysis, we focused on the following domains: play and fine motor (from BOQ for ages 0–4), upper extremity function and school re-entry (from BOQ for ages 5–18 and 11–18), and appearance and pain (all age questionnaires). These were chosen based on functional areas believed to be related to hand burns. Raw item responses within each domain were summed and converted to a standardized T-score based on normative data from non-burned children (BOQ 0–4) [21,25] or normative data from a sample of children with burns of less than 20% TBSA (BOQ 5–18 and BOQ 11–18) [24]. Domain scores are standardized with a mean of 50 and a standard deviation (SD) of 10. Scores that are at least one or more SD below the norm are considered to indicate clinically and socially relevant significant differences [25]. Each BOQ domain is scored independently, and results are interpreted within the context of pediatric recovery.

The PROMIS-UE Short Form 8a pediatric and proxy questionnaires were used for this study, which produce T-scores standardized to the general US pediatric population, with a mean of 50 and SD of 10. Higher scores indicate better UE function. A T-score of 50 represents average function in the general population. Prior work by Thissen et al. estimated minimally clinically important differences (MCID) across multiple pediatric domains to be 2–3 T-score points [28]. However, there is no established MCID specifically for the UE Short Form 8a. Scoring does not automatically adjust for age. Not all subjects followed up with each questionnaire at each time point and attrition rates are noted in Appendix A.

### 2.3. Sample Size Calculation

Based on a priori power analysis of functional outcomes related to burn severity as measured by HABS, a sample size of 90 participants would provide at least 90% power to detect a correlation as small as *r* = 0.30 between baseline HABS and 24-month outcomes at a two-sided significance level of 0.05. A correlation of this magnitude corresponds to approximately 9% of the variance in 24-month outcomes explained by HABS.

### 2.4. Statistical Analysis

**Figure 1:**
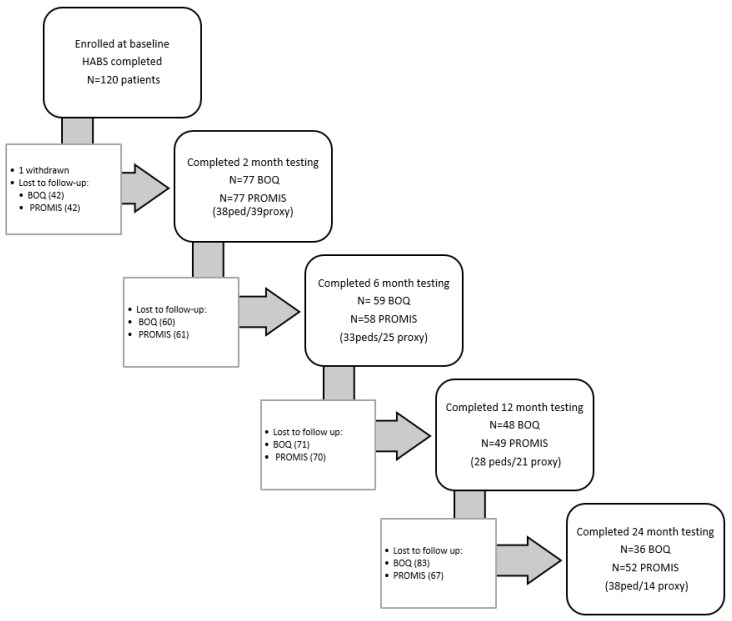
Patient flow diagram for subjects completing measures at each time point.

Clinical and demographic continuous variables were summarized as means and SD, or medians and interquartile ranges (IQR). Categorical variables were summarized as counts and percentages. A repeated-measures linear regression model was used to demonstrate how the interaction between patients’ hand burn severity, as indicated by their HABS score, and visit time was associated with the BOQ-UE domains and PROMIS-UE (pediatric and parent proxy) outcomes. For patients with bilateral hand burns, the higher HABS score (the score for the more severely burned side) was used. PROM outcomes were assessed at the patient level, while HABS is scored separately for each hand. Participants with bilateral burns, therefore, had two potential severity values. In these cases, the highest HABS score was used to represent severity. This approach was chosen to reflect the maximal functional impact of hand injury and to avoid within-subject correlation that would arise from modeling both hands separately. All models were adjusted for the following covariates: age, bilateral hand burn, hand surgery, and dominant hand burn. TBSA was not included in the final model because it was highly correlated with HABS scores, resulting in multicollinearity and unstable parameter estimates. Linear mixed-effects models were estimated using maximum likelihood, allowing inclusion of participants with incomplete follow-up data. This approach uses all available outcome observations (Figure 1) and provides a valid inference under the missing at random assumption. No separate imputation procedures were conducted. A compound symmetry covariance structure was used to account for the correlation between repeated measurements taken on the same patient over time. This study used the MIXED procedure with fixed and repeated measures in SAS version 9.4 (SAS Institute, Cary, NC, USA) to fit the repeated-measures linear regression models. All hypothesis tests were two-sided and evaluated at the 0.05 significance level.

### 2.5. Ethics

This study was reviewed and approved by Western Institutional Review Board (Protocol#20180571, Approved 14 March 2018). Written informed consent was obtained from a parent for children and adolescents under 18 years old, with additional assent for children and adolescents aged 8–17 years. Participants who were tested during follow-up visits after turning 18 years old were re-consented for continued participation.

## 3. Results

A total of 119 patients with 165 hand burns were enrolled in the study (Table 1). Patients were on average 8.5 years old (SD = 5.4), with a median age of 7.0 years (IQR: 4.0, 14.0). They were mostly male (56%), white (55%), and right-hand dominant (80%). Flame (54%), scald (25%), and contact (14%) burns were the most common etiology. Average TBSA was 12.7% (SD = 17.5%), and the median TBSA was 8.0 (IQR: 1.0, 17.5). Of the sample, 67% (*n* = 78) were admitted to the hospital for care, and 33% (*n* = 41) were seen only in the outpatient clinic. Of those admitted, the median length of stay (LOS) was 25.5 (IQR: 15.0, 36.0) days. Twenty-four patients were placed on mechanical ventilation, and the average number of ventilator days was 8.3 (SD = 8.8).

The dominant hand was burned in 69% of patients, and in 39% of cases, both hands were burned. Surgery was performed on 48 (40%) subjects and 69 (42%) hands. Of the hands that received surgery, the most common procedure was sheet split-thickness skin grafting (STSG), performed on 50 (30%) hands, followed by meshed STSG, performed on 11 (7%) hands. Four subjects and four hands underwent finger amputation of at least one digit. When considering the patients’ hands with the most severe injury HABS score, the median score was 4 (IQR: 2, 9, range: 1–18). Accounting for all HABS scores (including both hands for bilateral hand burns), the median score was 6 (IQR: 3, 12, range: 1–18).

### 3.1. BOQ Domains

**Figure 2:**
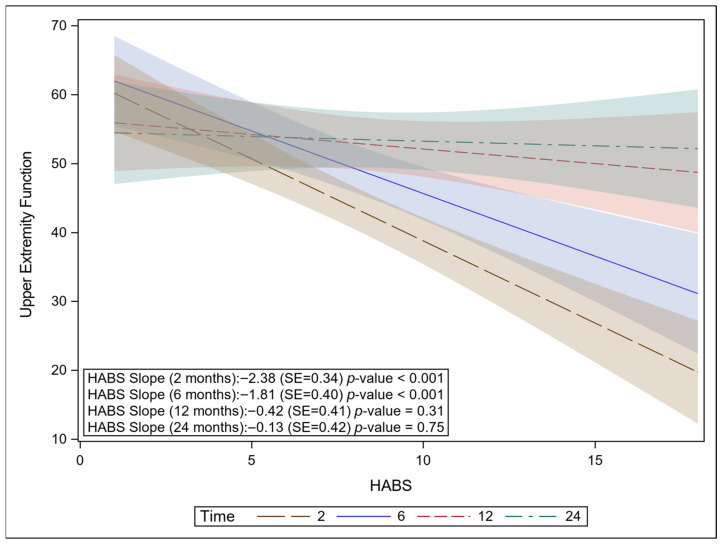
Negative association between HABS and BOQ-UE. As the HABS score increases, BOQ-UE significantly decreases at 2 months and 6 months. No association between the HABS score and UE function at 12 and 24 months.

HABS scores were significantly associated with the BOQ-UE function domain (*p* = 0.0019). As HABS increased (indicating more severe hand injury), UE Function decreased. There was a significant interaction between HABS and visit (*p* = 0.0004), indicating that the association between HABS and BOQ-UE differed across time points. At two-months post burn, a one-point increase in HABS score was associated with a 2.19 decrease in BOQ-UE score (*p* < 0.0001, 95% Confidence Interval [95% CI]: −3.04–−1.34) (Figure 2). At six months, the negative association persisted but was smaller. A one-point increase in HABS score was associated with a 1.53 decrease in BOQ-UE (*p* = 0.0021, 95% CI: −2.49–−0.57). By 12 months and 24 months, the association was no longer significant, indicating HABS was no longer a meaningful predictor of UE function at these later time points (*p* = 0.27 and 0.60, respectively). The analysis controlled for age, hand dominance, bilateral hand burn, and surgery. HABS was not significantly associated with any of the other BOQ domains evaluated, regardless of whether visit interactions were included.

Although HABS was not significantly associated with BOQ—Appearance in the multivariate analysis, patient age at the time of burn and having a dominant hand burned were both significantly associated with BOQ—Appearance scores. Increasing age (β = 0.58, SE = 0.23, *p* = 0.014) and dominant hand involvement (β = 6.76, SE = 2.96, *p* = 0.025) were associated with better perceived appearance. Outcome scores for each domain at each time point are available in Appendix A. HABS was not statistically associated with the BOQ—Pain domain; however, the mean Pain score at 2 months was greater than one SD from the standardized mean of 50, indicating clinical relevance. All other BOQ domains and visit times did not meet the BOQ criteria for clinical relevance. (Appendix A).

Evaluating HABS as a categorical predictor (HABS < 6 or HABS ≥ 6), as in other studies using HABS, we found it was not significantly associated with any of the BOQ domains.

### 3.2. PROMIS

**Figure 3:**
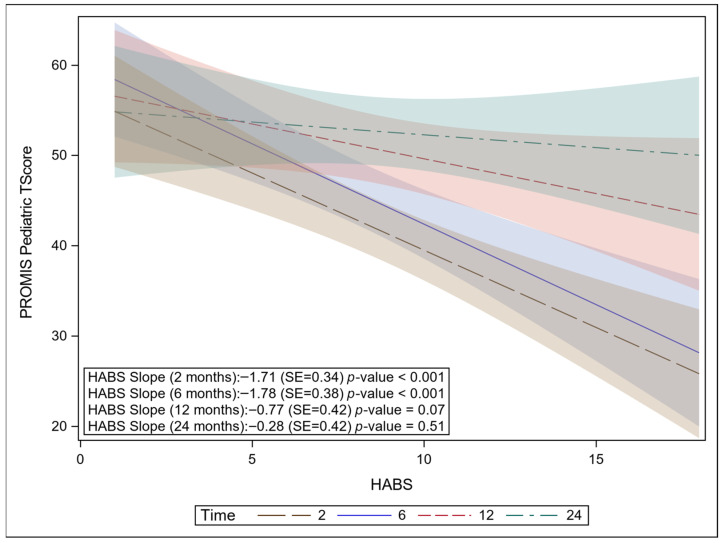
Negative association between HABS and PROMIS-UE8 pediatric. In an unadjusted model, the slope of HABS changes over time (significant interaction between HABS × time). As HABS increases, PROMIS UE-8 pediatric decreases at 2 months and 6 months. No association is observed between HABS and PROMIS at 12 and 24 months in this unadjusted model. This suggests that covariates correlated with HABS contributed to early functional limitations.

HABS was not a significant predictor of parent-reported PROMIS UE-8 scores (*p* = 0.55), indicating that HABS did not meaningfully influence how parents rated their child’s function over the course of follow-up. In contrast, HABS was a significant predictor of the patient-reported PROMIS UE-8 scores (β = −0.91, SE = 0.38, *p* = 0.02, 95% CI: −1.69–−0.15). In unadjusted analyses, the association between HABS and patient-reported PROMIS-UE8 varied over time, with stronger negative associations observed in the early post-burn period (Figure 3). However, after adjusting for covariates, the interaction with time was no longer significant (*p* = 0.12), and HABS remained consistently associated with patient-reported PROMIS-UE8 across all follow-up points. At each visit from 2 months to 24 months, mean T-scores for both patient and proxy PROMIS UE-8 scores improved by at least the MCID described by Thissen et al. (Appendix A).

## 4. Discussion

In this cohort of pediatric hand burn patients, higher HABS scores at the time of injury were associated with lower patient-reported upper extremity function, as measured by PROMIS-UE8, across the 2-year follow-up period. This finding indicates that more severe initial hand burn severity is linked with poorer perceived function. Using another clinical hand outcome measure, the BOQ, we observed a time-dependent relationship with scores in the UE function domain: higher HABS scores were strongly associated with poorer early BOQ-UE outcomes at 2–6 months post-injury, but the effect diminished over 12–24 months and was no longer significant. The attenuation of the association between HABS and BOQ-UE over time may reflect several non-mutually exclusive mechanisms. In addition to possibly reduced measurement sensitivity of the BOQ-UE, the lack of association at later time points may reflect that structural severity exerts a stronger influence on earlier functional outcomes, whereas healing, rehabilitation, and adaptation may reduce its impact over time. Additionally, differential attrition may have attenuated later associations, and therefore, the observed decline in association over time should be interpreted cautiously. These findings, however, underscore the importance of early interventions to optimize functional recovery in children with more severe hand burns, while recognizing that patients’ perceived limitations may persist beyond objective improvement.

The observed associations between hand burn severity and functional outcomes are consistent with, and build upon, prior burn literature emphasizing the impact of hand involvement on burn recovery in both children and adults. Prior studies using the BOQ have established that the presence of a hand burn increases the risk of functional and psychosocial challenges in pediatric burn survivors [4,27]. Palmieri et al. conducted a prospective study of children aged 5 years and younger and found that burns involving the hands, compared with those not involving the hands, were associated with greater deficits across multiple BOQ domains [4]. Similarly, Dodd et al. compared pediatric burn survivors 5–18 years old with and without hand involvement and demonstrated that BOQ-UE scores were lower at baseline for children with hand burns, and recovery was slower compared to children without hand burns [27]. Using the PROMIS-29 in an adult cohort, Thrikutam et al. found that hand burn severity was negatively correlated with UE function [28].

Our study supports these previous studies in demonstrating that patient-reported function is affected by hand burn involvement, using both the BOQ-UE function domain and the pediatric PROMIS-UE8 tool. However, our study advances previous findings by relating self-reported outcomes to hand burn severity using the HABS tool. Given the complex anatomy and function of the hands, the HABS allows for a more nuanced characterization of hand burn severity than using TBSA, which captures whole-body burn severity. Previous studies treated hand involvement as a binary variable (hand involvement or no hand involvement), which does not permit differentiation of risk within a spectrum of hand burns. Not all hand burns confer the same risk of poor outcome [13,14]. Our study used HABS scores as continuous variables, thus allowing us to overcome this limitation by quantifying the extent, depth, and location of the burn within the hand using HABS, which accounts for anatomy-specific considerations of burns to subregions of the hands that may differentially affect functional recovery.

Additional findings demonstrated that HABS was associated with child-reported PROMIS UE-8 outcomes but not with parent-reported PROMIS Proxy UE-8. This discrepancy likely reflects age- and perspective-related factors, including developmental differences in children’s self-perception of function and differences in measurement sensitivity between child- and parent-reported instruments, which is consistent with prior literature. This highlights the importance of incorporating both patient and parent perspectives in outcome assessment [26]. Parental assessment of function improved over time and, in this way, mirrored patient assessment; however, HABS was not significantly associated with parental scores. It is important to note that, for our study, either the patient or the parent completed the outcome measure based on the patient’s age; therefore, a direct comparison of scores was not possible.

The BOQ—Appearance domain was the only other BOQ domain with significant findings. While controlling for time since burn, HABS score, bilateral burn, handedness, and whether the patient had surgery, BOQ—Appearance scores were associated with patient age at the time of burn and dominant-hand burn. Older children and children with burns involving their dominant hand reported better self-perceived appearance. The protective effect of age and dominant-hand involvement may seem counterintuitive at first glance; however, this finding is supported by prior literature [29,30]. Pope et al. theorized that, unlike younger children, having the mental capacity, as a teenager, to confront and reason through body image challenges helps shape a more secure and positive patient-reported appearance score [30].

A similar line of reasoning might explain why burning the dominant hand was protective with respect to appearance scores. Not only is the dominant hand often the focus of intense rehabilitation, but a patient’s need to confront and adapt to a dominant-hand burn is much greater than that for a burn to the non-dominant hand, possibly leading to greater acceptance. We cannot rule out confounding in our methodology when interpreting this finding. Because parents completed the BOQ for patients under 8 years of age, this finding may reflect differences in perspectives between patients and their parents. Adolescents’ assessment of their appearance has been shown to be better than that of their parents in other studies [26].

The predictive value of HABS highlights its utility as a tool for triage and rehabilitation planning, helping clinicians identify which children are most vulnerable to prolonged upper extremity impairment. Early risk identification could help inform the types and timing of targeted surgical and rehabilitation interventions. For example, earlier referral to occupational and physical therapy, increased rehabilitation frequency or early implementation of splinting strategies. In addition, HABS scores can be used to inform families about recovery trajectories and help manage expectations of recovery.

This study has limitations inherent in its sample size and single-center design. Additionally, the sample sizes at the follow-up time points for some BOQ domains were small, limiting the ability to evaluate those functional categories. This study was conducted during the COVID-19 pandemic, which may have influenced participant follow-up and responses. Pandemic-related psychosocial stressors could not be isolated from the variables measured, which should be considered as future studies compare results. Attrition of follow-up at 12 and 24 months is likely affecting the association between HABS and BOQ-UE and PROMIS-UE. Larger sample sizes at those time points may have yielded statistically significant slopes, similar to those in the early time frames, thereby reinforcing the current findings for longer periods of follow-up. TBSA was highly correlated with HABS, and simultaneous inclusion in the model led to model instability. However, TBSA may still act as a confounder that warrants consideration in future studies. PROMIS and BOQ scores depend on patients’ subjective reporting and, like any self-report measure, may be influenced by familial, psychological, or sociocultural factors [3]. Further study is needed using objective functional measures. Another limitation of this study is that the amount and duration of rehabilitation provided to patients were not controlled. Lastly, MCID thresholds for BOQ-UE and PROMIS-UE have not been established, and our findings are reported using clinically meaningful differences found in available literature [21,31].

## 5. Conclusions

The BOQ-UE and PROMIS are two of the most widely used outcome measures for assessing pediatric burns [16,25]. Our findings reinforce the complementary strengths of these two measures for measuring outcomes in pediatric patients with hand burns. Using HABS as an indicator of severity to predict outcomes, both rounds out a comprehensive assessment of functioning when combined with these patient-reported outcomes measures and provides an expedient assessment analogous to TBSA, filling a gap in the assessment of hand function. The early identification of high-risk patients with hand burns, combined with rehabilitation and patient and family psychosocial support, may optimize both functional and quality of life outcomes. These results provide evidence for a more thorough model of evaluating recovery.

## Figures and Tables

**Table 1:** Sample Descriptive Statistics.

Subject Level Descriptors	Subjects (119)
	**Median [25th, 75th] (Range)**
Age (years)	7.0 [4.0, 14.0] (2.0–17.0)
TBSA (%)	8.0 [1.0, 17.5] (0.3–90)
Third-degree TBSA (%)	0.0 [0.0, 7.5] (0–90)
LOS for hospital-admitted subjects (days)	25.5 [15.0, 36.0] (1–222)
	**Frequency** ***n*** **(%)**
Male	67 (56%)
Surgery on at least one hand	48 (40%)
Admitted to the hospital	78 (67%)
Injured hand(s)	
Right	37 (31%)
Left	36 (30%)
Both	46 (39%)
Dominant Hand Burned	82 (69%)
Race	
White	65 (55%)
Black	6 (5%)
Native Hawaiian/Pacific Islander	0
Asian	2 (2%)
American Indian or Alaskan Native	2 (2%)
Unknown	3 (3%)
Missing	36 (30%)
Other	5 (4%)
Burn Etiology	
Scald	30 (25%)
Flame	64 (54%)
Contact	17 (14%)
Chemical	1 (1%)
Friction	6 (5%)
	**Median [25th, 75th] (range)**
Hand with more severe injury HABS score	4 [2, 9] (1–18)
	**Frequency** ***n*** **(%)**
HABS < 6	65 (55%)
HABS ≥ 6	54 (45%)
Hand-level descriptors	**Hands (165)**
	**Frequency** ***n*** **(%)**
Type of surgery	
None	96 (58%)
STSG, sheet	50 (30%)
STSG, mesh	11 (7%)
FTSG	7 (4%)
Flap	1 (1%)
Finger amputation	4 (2%)

FTSG = full-thickness skin graft; HABS = Hand Burn Severity Score; LOS = length of hospital stay; STSG = split-thickness skin graft; TBSA = total body surface area.

## Data Availability

The data presented in this study are available on request from the corresponding author due to privacy restrictions. Access to the raw de-identified data supporting the conclusions of this article may be granted upon reasonable request to the corresponding author on a case-by-case basis with the approval of Shriners Hospitals for Children compliance and legal departments.

## Abbreviations

The following abbreviations are used in this manuscript:

BHOT	Burnt Hand Outcome Tool
BOQ	Burn Outcomes Questionnaire (full questionnaire)
BOQ-UE	Burn Outcomes Questionnaire Upper Extremity Domain
HABS	Hand Burn Severity Score
IQR	interquartile range
MCID	minimally clinically important difference
PROMIS	Patient Reported Outcomes Measurement Information System
PROMIS-UE8	Eight-item Patient-Reported Outcomes Measurement Information System-Upper Extremity
SD	Standard Deviation
TBSA	Total Body Surface Area

## Appendix A

**Table A1:** Outcome Scores at Each Time Point.

BOQ Domain Scores at Each Time Point and Scar Age at the Visit.				
Visit Month (Total *n*)	2 (*n* = 78)	6 (*n* = 57)	12 (*n* = 46)	24 (*n* = 35)
	Median (IQR)	Median (IQR)	Median (IQR)	Median (IQR)
Scar Age at visit (days)	46.0 (35.0, 60.0)	186.5 (157.0, 219.0)	366.0 (343.0, 402.0)	734.0 (712.0, 744.0)
BOQ Domains	Mean (SD)	Mean (SD)	Mean (SD)	Mean (SD)
Upper Extremity Function **	44.2 (19.33) (*n* = 56)	49.4 (14.53) (*n* = 43)	52.1 (5.68) (*n* = 36)	53.1 (4.17) (*n* = 32)
School Reentry **	50.1 (11.37) (*n* = 34)	53.2 (11.38) (*n* = 35)	50.7 (11.09) (*n* = 33)	53.9 (11.55) (*n* = 34)
Play *	51.5 (7.16) (*n* = 21)	52.4 (8.39) (*n* = 12)	51.3 (8.48) (*n* = 10)	56.3 (0.00) (*n* = 2)
Fine Motor *	48.2 (9.67) (*n* = 21)	55.3 (4.63) (*n* = 14)	55.1 (6.18) (*n* = 10)	58.6 (0.00) (*n* = 2)
Appearance	48.0 (14.51) (*n* = 76)	50.4 (13.76) (*n* = 57)	49.7 (12.70) (*n* = 45)	51.1 (10.58) (*n* = 35)
Pain	39.8 (17.94) (*n* = 77)	48.1 (16.57) (*n* = 56)	47.7 (15.55) (*n* = 45)	51.1 (10.14) (*n* = 34)
**PROMIS-UE8 scores at each time point.**				
**Visit Month**	**2** **(*n* = 79)**	**6** **(*n* = 60)**	**12** **(*n* = 49)**	**24** **(*n* = 52)**
	Mean (SD)	Mean (SD)	Mean (SD)	Mean (SD)
T score PROMIS proxy	34.8 (12.35) (*n* = 39)	38.0 (11.34) (*n* = 25)	41.2 (13.30) (*n* = 21)	46.3 (10.37) (*n* = 14)
T score PROMIS pediatric	42.1 (14.92) (*n* = 38)	46.0 (13.38) (*n* = 33)	50.3 (9.45) (*n* = 28)	52.8 (7.41) (*n* = 38)

* BOQ 0–4 only; ** BOQ 5–18 and BOQ 11–18 only; Scar age is calculated as the time from burn (days).
